# Professionalization in the public sector health supply chain management: IAPHL’s present and future contribution

**DOI:** 10.1186/2052-3211-7-S1-O13

**Published:** 2014-12-17

**Authors:** Lea Teclemariam, Chris Wright

**Affiliations:** 1 International Association for Public Health Logisticians (IAPHL)/John Snow Inc., Washington DC, USA; 2 International Association for Public Health Logisticians (IAPHL)/John Snow Inc., Addis Ababa, Ethiopia

## Background

In the 1990s, public sector technicians, mainly pharmacists, were gaining skills to fulfil their responsibilities in supply chain management (SCM) through different training sessions. Supply chain management was neither recognized as a unique profession nor institutionalized under Ministries of Health. The increasing need for global dialogue regarding health supply chain experiences, skills, and best practices was evident. To respond to this need and to strengthen the professionalization of supply chain management, the International Association of Public Health Logisticians (IAPHL) was created in 2007.

## Method

IAPHL provides a free membership association for logisticians to support one another by sharing information, experiences, and resources through an online listserv. Members participate in online discussions led by technical experts on topics suggested in the annual member satisfaction survey. Other than these community driven discussions and resources, IAPHL has also sponsored members to the annual Global Health Supply Chain Summit conference, where they had the opportunity to hear different ideas from academicians, researchers and practitioners.

## Results

The membership of the association has grown from 120 in October 2007 to 2656 in June 2014 in 114 countries, with increasing member engagement. Members have been actively participating in the discussions on the listserv, and in the past year alone the association has received 50 or more contributions per month consistently for 10 out of the 12 months.

Out of the 160 respondents to the 2014 annual survey, 73% reported that the association has increased their SCM knowledge.

## Discussion

The main goal of the association is engaging existing members and attracting a diverse group of new members. Consequently, expanding the portfolio of professional development activities and maintaining the quality of these services remain at the core of the association.

The results show that IAPHL has brought recognition and made contribution towards professionalization of health SCM in the public sector. Future contribution to professionalization will involve resolving a number of questions such as the measurement of the effectiveness of its professional development activities, sustainability and inclusion of potential non-English speaking members.

## Lessons learned

Professional associations such as IAPHL can be great vehicles for promoting professionalization of public sector health supply chain managers and building their professional capacity to improve supply chain performance in their countries. Increased investment should be made to bolster such associations to ensure they provide services to shape the future of supply chain management, especially in the public sector.

